# Empowering Obese Children in Physical Education: Exploring the Influence of Verbal Encouragement on Games Intensity, Mood States, and Physical Enjoyment during Passing Games

**DOI:** 10.1371/journal.pone.0319414

**Published:** 2025-04-16

**Authors:** Bilel Aydi, Okba Selmi, Bruno E. Figueira, Mohamed Abdelkader Souissi, Bruno Gonçalves, Katsuhiko Suzuki, Beat Knechtle, Che-Yi Yang, Yung-Sheng Chen, Nizar Souissi

**Affiliations:** 1 High Institute of Sports and Physical Education of Kef, University of Jendouba, El Kef, Tunisia; 2 Research Unit, Sportive Sciences, Health and Movement, El Kef, Tunisia; 3 Departamento de Desporto e Saúde, Escola de Saúde e Desenvolvimento Humano, Universidade de Évora, Évora, Portugal; 4 Comprehensive Health Research Centre (CHRC), Universidade de Évora, Évora, Portugal; 5 Physical Activity, Sport and Health, Research Unit, National Observatory of Sport, Tunis, Tunisia; 6 High Institute of Sports and Physical Education of Gafsa, University of Gafsa, Gafsa, Tunisia; 7 Faculty of Sport Sciences, Waseda University, Tokorozawa, Japan; 8 Institute of Primary Care, University of Zurich, Zürich, Switzerland; 9 Medbase St. Gallen Am Vadianplatz, St. Gallen, Switzerland; 10 Department of Physical Education, Fu Jen Catholic University, New Taipei City, Taiwan; 11 High Performance Unit, Chinese Taipei Football Association, New Taipei City, Taiwan; 12 Department of Exercise and Health Sciences, University of Taipei, Taipei, Taiwan; 13 Exercise and Health Promotion Association, New Taipei City, Taiwan; 14 High Institute of Sport and Physical Education, Ksar-Saïd, Manouba University, Tunis, Tunisia; Sorbonne Paris Nord University, FRANCE

## Abstract

This study aimed to assess the impact of physical education teachers’ verbal encouragements on the psychophysiological aspects, physical enjoyment, and mood states of obese children (OC) in a physical education context. Sixteen OC students (mean age = 13.81 ± 0.73 years) from a preparatory school participated in two test sessions, conducted in a randomized order. Each session involved a series of passing games (games of 10 successive passes) with and without verbal encouragement, with a one-week interval between sessions. The games, formatted as 3 vs. 3 with two additional obese joker’ players, lasted 18 minutes. Each game comprised four 3-minute active periods interspersed with 2-minute passive recovery bouts, played on a 10 × 20-m pitch. Heart rate was continuously measured throughout each session. Additionally, the Brunel Mood Scale (BMS) was assessed before and after the PG. Furthermore, rating of perceived exertion (OMNI-RPE) and physical activity enjoyment (PACES) were assessed after the testing sessions. Video analysis was used to quantify technical actions during PG. PGs with VE induced higher HR (% maximum HR and mean HR), OMNI-RPE, and PACES scores than PGs without VE (ES=−1.51, ES=−0.78, ES=−0.73, ES=2.07, respectively). Compared with PGs without VE, SSGs with VE resulted in an increased percentage of successful passes, number of Goal (10 passes) and fewer lost balls (ES=O.70, ES=−0.54, ES=−0.86, respectively). The PGVE trial also showed higher vigor and lower total mood disturbance (TMD) compared to the PGNVE trial (ES = −1.11, d = 0.78, respectively). Physical education teachers are encouraged to incorporate joker exercises with verbal encouragement to enhance game intensity, mood state, physical enjoyment and technical performance during games among OCs.

## Introduction

Childhood obesity has emerged as a critical public health issue worldwide, with its increasing prevalence leading to far-reaching consequences that impact medical, psychological, and socio-emotional domains [1,2]. This condition is not merely a matter of excess weight; it is associated with serious health risks, including cardiovascular diseases, type 2 diabetes, and various musculoskeletal complications, even in young children [3]. Beyond these physical health challenges, obese children (OC) often grapple with psychological and emotional difficulties, such as heightened anxiety, low self-esteem, persistent fatigue, and negative body image. These issues are exacerbated by social stigma and weight-based discrimination, significantly hindering their active participation in physical education (PE) classes [4].

In the school environment, negative stereotypes frequently marginalize OC, portraying them as unmotivated or less competent. This perpetuates a cycle of exclusion and disengagement [4–7]. Such erroneous perceptions contribute not only to their difficulties in engaging with physical and social activities but also intensify their vulnerability to weight-based bullying, a major source of dissatisfaction with PE programs that detrimentally impacts the psychological well-being of these children [8]. Consequently, negative experiences further diminish their intrinsic motivation to participate actively in sports and exercise, creating barriers to a healthy lifestyle.

Given these significant challenges, it is imperative for schools to establish inclusive, supportive initiatives that specifically address the unique needs of OC in PE activities. However, physical education teachers (PETs) often encounter obstacles, as these children typically exhibit lower levels of physical activity, reduced stamina, and weaker motor skills [9,10]. Recognizing and addressing these specific requirements is crucial for PETs, enabling them to design activities that foster participation and positive experiences for OC. Without tailored approaches, the cycle of exclusion is likely to continue, perpetuating negative outcomes for these vulnerable students.

Recent studies highlight the importance of providing obese children with supportive interventions that improve not only their physical fitness but also their socio-emotional resilience. Game-based teaching methods, such as the Teaching Games for Understanding (TGFU) model, have emerged as particularly effective in PE contexts. TGFU focuses on enhancing student understanding and engagement through enjoyable and inclusive gameplay, helping students develop technical abilities and tactical skills while promoting positive emotions [11,12]. This approach is especially relevant for OC, as it emphasizes enjoyment and participation over rigid performance metrics, fostering an environment where they can thrive without the pressure of traditional fitness expectations.

To effectively implement TGFU, it is crucial to understand how obesity is defined among participants and whether specific assessments were conducted to accurately categorize children as obese. Detailed information regarding the criteria for obesity, including body mass index (BMI) measurements or other relevant health evaluations, should be provided. Furthermore, the exercise history of the children should be considered, as this can influence their engagement levels and the appropriateness of the chosen activities.

Incorporating group game activities, particularly passing games (PGs), into physical education curricula has been shown to foster enthusiasm, integration, and communication among students [12,13]. These games not only engage students physically but also create a supportive environment where they can interact socially, which is crucial for reducing feelings of isolation among OC. By prioritizing gameplay context, TGFU encourages students to grasp underlying strategies and concepts rather than merely focusing on technical skills. This game-centered approach immerses them in the excitement and challenges of play, making learning more relevant and enjoyable. Additionally, the choice of play-based activities over motor skill-based ones is intentional; the former is more likely to promote participation among OC by emphasizing social interaction and enjoyment rather than technical skill development. This is particularly important, as the aim is to encourage active participation rather than to emphasize competition or performance.

Research also demonstrates that coach encouragement significantly influences young athletes’ psychophysiological responses and performance in various sports contexts. Studies indicate that supportive coach feedback enhances engagement and performance in activities like small-sided soccer games [13,14] and tennis. Specifically, the work by [15] highlighted the effects of coach encouragement on psychophysiological responses and technical actions in different small-sided soccer games [16]. Additionally, [17] examined the impact of coach encouragement on the psychophysiological and performance responses of young tennis players [18]. This role of verbal encouragement from PETs is especially pertinent for OC, as it can bolster their motivation, enjoyment, and engagement in physical activities. When PETs provide verbal encouragement throughout PE classes, students tend to exert more effort, experience improved self-perception, and enjoy physical activities more [19]. Conversely, a passive approach lacking verbal encouragement can detrimentally affect the mood and enjoyment of OC [20,21].

Verbal encouragement fosters a supportive atmosphere, empowering OC to participate actively and helping them overcome self-doubt [22,23]. Moreover, it nurtures a sense of belonging and acceptance, alleviating feelings of isolation and anxiety often experienced by these children during physical activities [24]. Thus, verbal encouragement acts as a catalyst that boosts morale, enhances enjoyment, and promotes sustained participation in physical activity among OC [25]. Furthermore, [15] investigated how coach encouragement affects soccer test performance, emphasizing its significance in enhancing performance outcomes [19].

Despite existing research on the effects of verbal encouragement, there remains a gap in understanding how specific encouragement techniques, particularly those derived from the TGFU approach, can be integrated into PE programs for OC. The TGFU model emphasizes enhancing student engagement through enjoyable gameplay, which aligns with the needs of OC by promoting a non-competitive atmosphere. Additionally, it is crucial to clarify how obesity is defined among participants and whether any tests or analyses were performed to ensure accurate group assignments, considering the children’s exercise history and the rationale for choosing play-based activities over motor skill-based ones.

This study aims to address these gaps by assessing the effect of PET’s verbal encouragement on the psychophysiological aspects, physical enjoyment, and mood states of OC during passing games (PGs). Specifically, this research seeks to determine how positive verbal interactions during exercise can improve the well-being and engagement of OC in physical activities, providing insights for better integration of verbal encouragement into PE programs tailored for this population.

## Methods

### Participants

Sixteen male OC from a preparatory school were recruited for this study (Mean age = 13.81 ± 0.73 years, height = 162 ± 4.18 cm, body mass = 79.75 ± 3.75 kg, fat = 30.56 ± 0.53%). The participants have a minimum of 7 years of experience in physical education, including 6 years in primary school and at least 1 year in middle school. During this time, they have practiced various individual sports, such as athletics and gymnastics, as well as several team sports, including handball, basketball, volleyball, and football’‘. These students were enrolled in the 7th and 8th grades of basic education and participated in two weekly PE session, for a total of 3 hours. The sample size determination was conducted using the G*Power software, version 3.1. Based on a Cohen’s d effect size of 1.08, as reported in a similar study by [26], with a significance level set at α = 0.05 and a statistical power of 0.95 (1-β), the calculation indicated that 11 participants were required. The study took place during their scheduled PE classes. Inclusion criteria were as follows: (1) all students attended the same school, (2) they were under the instruction of the same PET, (3) no history of injury or illness was reported during the two months prior to the study, and during the study period, (4) lack of known physical or cognitive disorders, and (5) students with a body mass index (BMI) exceeding 30 kg/m^2^. Exclusion criteria included: (1) irregular attendance in PE classes, and (2) occurrence of illness during the experimental period. Both students and their parents voluntarily provided written consent after receiving detailed explanations about the research objectives and associated risks. This study was conducted in accordance with the latest version of the Declaration of Helsinki, and the protocol was fully approved by the Research Ethics Committee of the High Institute of Sports and Physical Education of Kef, Jendouba, Tunisia (approval no. 012/2021) prior to the commencement of data collection. The study period was from January 8, 2023, to January 28, 2023, which included 1 week for familiarization and the execution of anthropometric measurements and the Vameval test, followed by 2 weeks dedicated to the experimental protocol.

### Procedures

Prior to the experimental sessions, anthropometric measurements were conducted by trained researchers, and participants completed the VAMEVAL test to estimate maximum heart rate (HRmax) [13]. This study investigated the psychophysiological response, mood state, and enjoyment of OC during PG based on handball rules (games of 10 successive passes) under two conditions: PG with VE and PG without VE. Two OCs participated as Joker players in each game, while the remaining players were children of normal weight (NC). The two PG sessions were conducted one week apart during regular physical education (PE) classes.

During each experimental week, the Participants were divided into two equal groups of eight. The groups were assigned in a randomized and counterbalanced manner to ensure unbiased results and to control for any potential order effects. Eight OCs completing PG with VE (PGVE) and another group of eight completing PG without VE (PGNVE). This design allowed each participant to experience both conditions: PGVE once and PGNVE once.

Before and after each trial (PGVE and PGNVE), the mood state of participants was assessed using the Profile of Mood States (POMS), while heart rate (HR) was continuously monitored throughout each session. Additionally, Ratings of Perceived Exertion (RPE) and the Physical Activity Enjoyment Scale (PACES) were recorded immediately after each trial. Participants independently completed the scales to minimize peer influence. To minimize learning effects, all OCs were familiarized with the RPE, PACES, POMS questionnaire, and the PG protocol before the start of the experimental sessions. All measurements were conducted in the same school indoor sports hall during regular PE classes. Data for each test session were collected by the same physical education teacher (PET).

### Passing games session

A standardized 12-minute warm-up session was conducted for all students, including both OC and those with normal weight who were participating in the PE session. This warm-up routine incorporated elements such as jogging, proprioception exercises, dynamic stretching, and coordination movements, culminating with a series of 3 sets of 10-meter accelerations. Following the warm-up, a three-minute period of passive recovery was provided before the commencement of the first PG bout. The PG took place on an outdoor field, adhering strictly to the 3 vs. 3 + 2 obese Joker play format observed in previous studies [14]. Specifically, each team consisted of three NC players alongside two obese Joker players (OC), who alternated between both teams. This methodological choice was motivated by the goal of ensuring active participation from the obese students, enabling them to touch the ball more frequently. By facilitating their interaction with the game, we aim to enhance their concentration and engagement, which are essential for their physical and social development. The pitch dimensions were set at 20 × 10 meters, providing approximately 25 square meters of playing space per player. Each PG trial session lasted 18 minutes, comprising four bouts, each lasting 3 minutes, separated by 2 minutes of passive recovery [14]. Points were awarded to the team that completed 10 successive passes. Following each bout, the losing team faced sanctions imposed by the PE teacher, involving 10 repetitions of exercises such as push-ups, setups, or squats. Each obese Joker player was required to make at least one pass during every sequence of 10 successive passes for the point to be awarded. During PGVE, the PE teacher (PET) actively moved around the field perimeter, offering targeted verbal encouragement (VE) specifically directed at obese children to maximize their engagement and participation in the game. This support was not random; it was spontaneous and based on observable elements of the game situation. The instructors considered each child’s position on the field, their movements, spatial awareness, and ball circulation. Through this approach, the PET provided game-specific cues such as “Go Go Go,” “Again Again,” “attack the ball,” “seek the ball,” and “keep the ball” [13,27]. The PET also supplied new balls as needed to sustain continuous play, using spontaneous encouragement in response to the dynamics of the game. This method aimed to create an encouraging environment that promoted not only the development of children’s technical skills but also their motivation to actively participate in the game. In PGNVE, however, the PET remained beside the field, supplying new balls as needed but refraining from any VE. Players were instructed to give maximum effort and attempt to maintain possession of the ball. To ensure equal participation, each player was restricted to three steps with the ball throughout the PG session.

### Measures

#### Anthropometric measurements.

Anthropometric measurements of height and body mass were taken from the subjects whit subjects barefoot and wearing only lightweight shorts. Height measurements were recorded to the nearest 0.1 cm using a Harpenden stadiometer.

During height measurements, OC stood upright with their heels together, arms extended, and head in a parallel position to the floor. Body mass measurements were to the nearest 0.1 kg, using an electronic scale (Tanita, Model TBF-410 GS, Tokyo, Japan). During this process, subjects stood upright at the center of the scale platform, with arms extended to the sides. Body Mass Index (BMI) was calculated using the formula: weight (kg)/ height (m)^2^ (BMI = weight (kg)/ height (m)^2^).

### VAMEVAL test for maximum heart rate evaluation

All children underwent the VAMEVAL test to determine individual maximum heart rate (HRmax). The evaluation was conducted on a 200 m running track following the protocol outlined by [13]. Participants followed a pre-programmed auditory cue (a beep) while running, with ten cones spaced every 20 m along the track. The initial running speed was set to 8 km/h, with increments of 0.5 km/h every minute until participants reached exhaustion. Participants adjusted their pace based on the position of the cones. The test concluded when a participant failed to maintain the required speed for two consecutive intervals or felt unable to continue. HR was monitored using a Polar Team Sport System (Polar-Electro OY, Kempele, Finland), and the highest average value over a 5-second period during the test was recorded as VAMEVAL HRmax.

### Assessment of technical demands in passing games

The percentage of successful individual passes, the number of individual lost balls, the number of individual interceptions, and the number of goals scored (per 10 passes) were among the technical standards that were evaluated after each PG. Sony Handy-cam DVD 850 cameras were set up at an elevated vantage point along each pitch’s halfway line to film the PGs on video [13,28]. Technical data was manually recorded using notation that followed Table 1 definitions. Every PG video was examined twice by a single, highly qualified researcher in order to assess intra-rater consistency using the kappa coefficient [13,28]. The evaluated variables’ kappa values varied from.91 to.97, demonstrating a high degree of intra-rater reliability.

**Table 1:** Definitions of Skills Employed in this Research.

Technical variable	Definition
Successful passes	Transferring the ball from one player to another successfully shows efficient ball distribution and possession retention.
Lost balls	Situations in which a team gives up the ball because of an incomplete throw, an opponent’s interception, or an out-of-bounds play that leads to a turnover.
Interceptions Goals	Defensive plays in which a player from the other team intercepts the ball and keeps it from going to the intended recipient, disrupting a pass attempt.
Scored (per 10 passes)	Offensive efficiency metric shows how well passes create scoring opportunities by expressing the number of goals scored in relation to every 10 passes attempted.

### Assessment of perceived exertion

Following each intervention immediately (PGVE and PGNVE), the children’s internal intensity was evaluated using the OMNI-RPE (0–10) scale [29]. This scale assesses the subjective intensity of exercise by asking participants, “How was the exercise and how did you feel?” The OMNI scale spans from 0 to 10, encompassing a spectrum from “Extremely Easy”(0) to “Extremely Hard” (10). Descriptive terms and accompanying images aid in the assessment of perceived exertion during exercise sessions. These visual aids enhance participants’ understanding of the numerical RPE values. The validity of this perceived scale has been established through its utilization in various scientific studies [30,31,32].

### Assessment of mood states

For monitoring mood state variations during training exercises, we utilized the 24-item Brunel Mood Scale (BMS). The scale was administered to the children both before and after each PG intervention to assess six emotional states: vigor, fatigue, depression, confusion, tension, and anger. Each item on the BMS is rated on a 5-point scale, ranging from 0 (indicating “Not at all”) to 4 (indicating “Extremely”). The Total Mood Disturbance (TMD) score, reflecting overall mood disturbance, was calculated using the formula: TMD = (Fatigue + Anger + Confusion + Tension + Depression) – positive mood (Vigor) + 100. To prevent negative scores, 100 was added to the total score. In the current study, the Cronbach’s α coefficient for the BMS ranged from 0.87 to 0.93.

### Assessment of enjoyment

Five minutes after completing both PGVE and PGNVE, children completed the physical activity enjoyment scale (PACES) to assess their enjoyment levels [33]. Children were asked to assess “how they felt at the moment about the physical activity they had been engaging in.” The PACES inventory consists of 8 items, each rated on a 7-point scale ranging from 1, “It is very pleasant,” to 7, “It’s not fun at all.” The total physical enjoyment score was computed by summing the scores of the 8 items, resulting in possible ranges from 8 to 56 points. Higher scores indicate greater levels of enjoyment experienced during the physical activity. The Cronbach’s α coefficient for the PACES test in this study was 0.91, indicating a high level of internal consistency.

### Physiological assessment during passing games (PG)

The maximum heart rate (HRmax) was estimated using the VAMEVAL test, with HRmax identified as the highest heart rate reached during the test. To assess exercise intensity during PGs, heart rate (HR) was monitored every 5 seconds using the Polar Team 2 Pro System by Polar Electro OY. Participants maintained consistent positioning of the HR sensor throughout the physical activity. Subsequently, HR data were expressed as a percentage of VAMEVAL-HRmax (%HRmax) and mean HR (HRmean). HRmean for PGVE and PGNVE was calculated. The %HRmax for each type of training session was determined using the formula: %HRmax = (HRmean/ VAMEVAL-HRmax) * 100 [26].

### Statistical analysis

The verification of normality and sphericity assumptions preceded the use of parametrical statistical procedures. The statistical analysis employed in this study involved the use of the paired student t-test to compare conditions. Additionally, Cohen’s d was computed as the effect-size measure. The predetermined alpha level for all statistical tests was p = 0.05, and the computations were conducted using IBM SPSS Statistics for Windows, version 24.0 (IBM Corp., Armonk, NY). The established benchmarks for effect size statistics were as follows: less than 0.2, considered trivial; less than 0.6, classified as small; less than 1.20, categorized as moderate; less than 2.0, deemed large; and greater than 2.0, considered very large.

## Results

**Fig 1:**
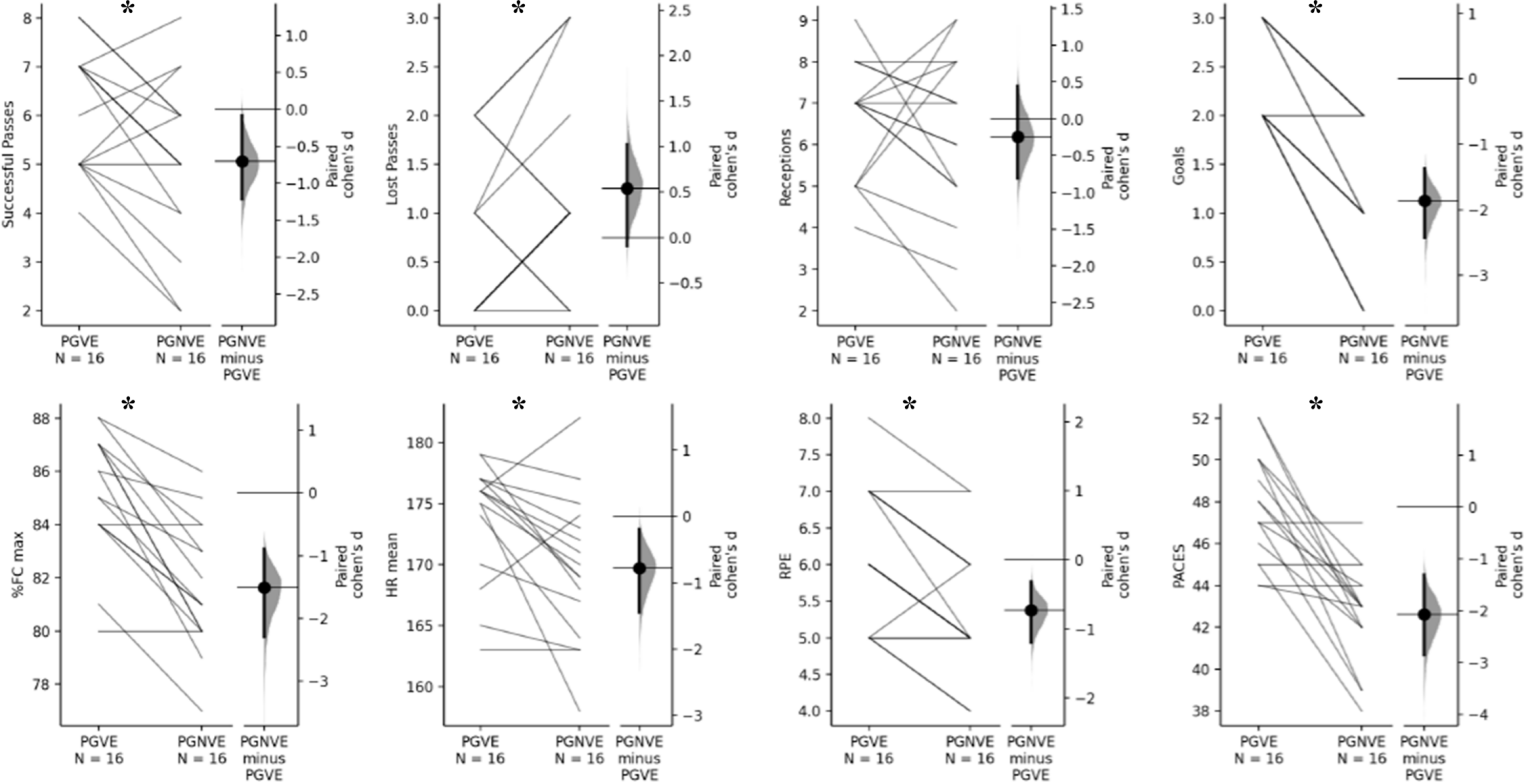
The technical performance, psychophysiological responses, and enjoyment between PGVE and PGNVE trials. p= Between group-subject effect; *= indicates significant differences between conditions; PGVE = passing game with verbal encouragement; PGNVE = passing game without verbal encouragement; ES = effect size; Suc. Pass= Successful passes; RPE = rated of perceived exertion.

**Fig 2:**
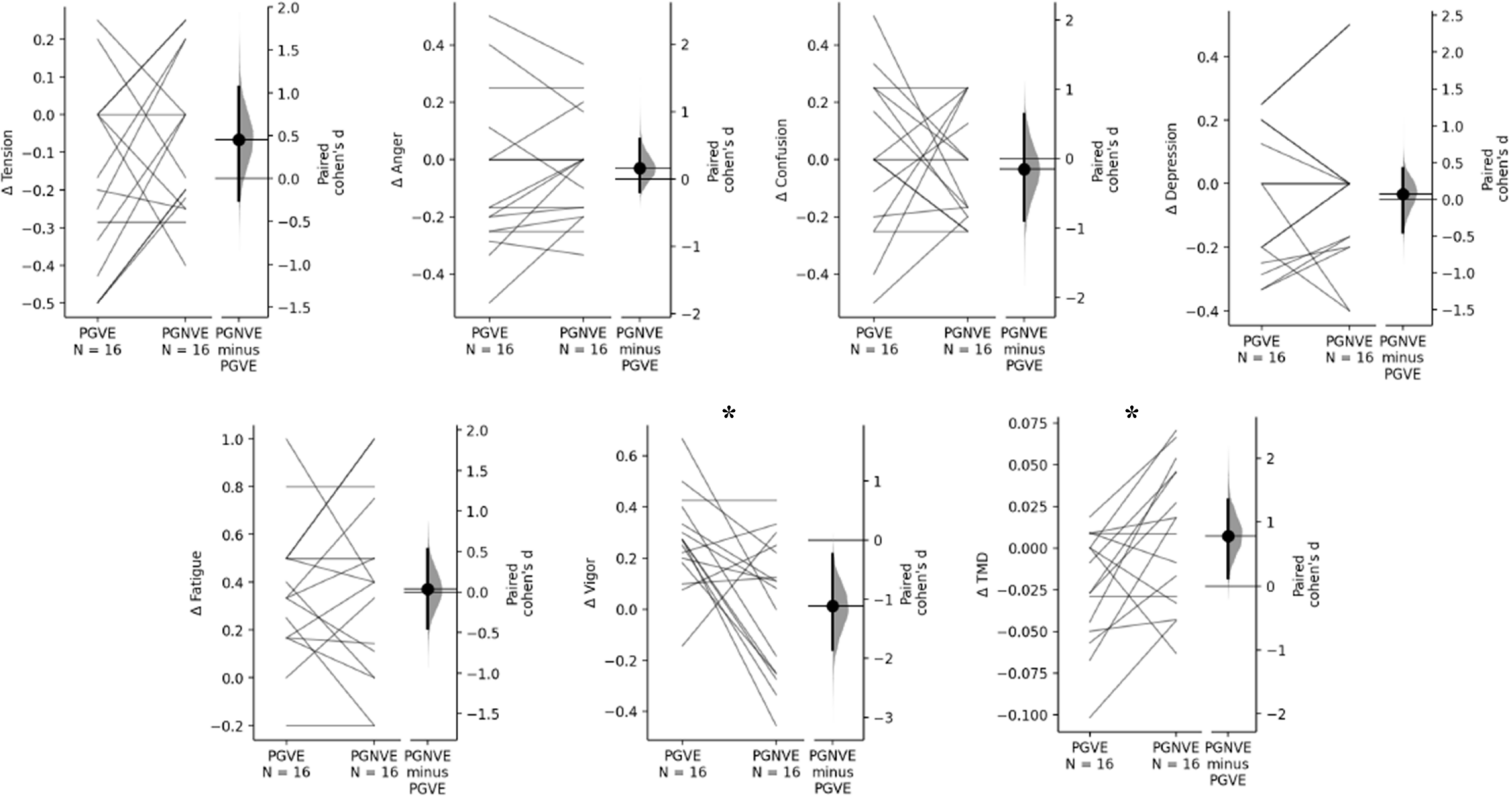
The results of mood status between PGVE and PGNVE trials. p = Between group-subject effect; *= indicates significant differences between conditions; PGVE = passing game with verbal encouragement, PGNVE = passing game without verbal encouragement; TMD = Total Mood Disturbance.

The performance variables showed differences between PGVE and PGNVE conditions; successful passes (p=0.01, ES=0.70); lost passes (p=0.03, ES=−0.54); Goals (p=0.00, ES=**−**1.86); %Fc max (p=0.00, ES=**−**1.51); HR mean (p=0.01, ES=**−**0.78); RPE (p=0.00, ES=**−**0.73); PACES (p=0.00, ES=2.07); ΔVigor (p=0.01, ES=**−**1.51); and ΔTMD (p=0.02, ES=0.78) (Fig 1 and Fig 2). In opposition, the comparison between both conditions presented no significant effect on reception, tension, anger, confusion, depression and fatigue (p>0.05, no effect).

## Discussion

The present study sought to explore the impact of the physical education teacher’s verbal encouragement (VE) on game intensity, mood state, and physical enjoyment among obese children during physical games (PGs). The findings revealed that: (1) PGs with VE led to higher mean heart rate (HR), percentage of HRmax, and internal intensity (OMNI-RPE) compared to PGs without VE; (2) participants reported greater physical enjoyment (PACES score) following PGs with VE; and (3) PGs with VE elicited a more positive mood state compared to those without.

The role of verbal encouragement (VE) in enhancing psychological aspects such as confidence and motivation is important, particularly in the context of obese children who may face additional barriers to engagement in physical activity [34]. Research has shown that a supportive environment created by PET can significantly influence a child’s self-perception and motivation to participate actively [35,36]. By fostering a positive emotional climate through VE, PET can help children feel more competent and valued, which in turn may enhance their willingness to engage in physical games [37]. This aligns with Self-Determination Theory, which posits that fulfilling psychological needs—such as competence and relatedness—promotes intrinsic motivation [38]. Thus, while confidence and motivation were not directly assessed in our study, the observed improvements in intensity, enjoyment, and mood state suggest that VE could play a significant role in building these critical psychological factors [26,39,40].

In terms of psychophysiological responses, this study demonstrated a significant increase in psychophysiological indicators during PGs with VE, indicating heightened physical demands and effort exerted by the obese children (OC) [34]. The presence of VE correlated with higher mean HR, percentage of HRmax, and OMNI RPE, suggesting that a supportive coaching environment motivates children to push their limits, enhancing their effort and commitment to exercise. These findings underscore the importance of VE in intensifying gameplay, as evidenced by increased OMNI RPE scores and HR during PGs with VE. Consistent with prior research, VE has been shown to elevate physiological responses and internal intensity during physical activity. For instance, [17] demonstrated the positive effects of coach encouragement on the psychophysiological and performance responses of young tennis players [41]. Similarly, [15] reported that VE significantly influenced psychophysiological responses and technical actions in small-sided soccer games [42]. Together, these studies reinforce the notion that motivation provided by teachers fosters greater engagement and commitment, promoting maximum-effort training among OCs.

In relation to technical performance, PGs are utilized to enhance participants’ physical condition and technical skills. This study demonstrates that VE positively impacts technical performance during PGs, marking the first such evidence among OCs. Specifically, OCs achieved more successful passes, scored more goals (10 passes), and made fewer mistakes during PGs with VE. The use of this motivational technique from physical education teachers (PET) can thus enhance technical performance in OCs. This finding aligns with previous research, which emphasized the positive influence of VE on participants’ technical and tactical actions during small-sided games [23]. Furthermore, studies by Kilit et al. and Soylu et al. support the argument that VE enhances both psychophysiological responses and technical actions in various sports contexts [41,42]. These results suggest that external motivation, particularly through verbal encouragement, enhances concentration during gameplay, leading to improved ball possession and increased precision throughout the games. Consequently, motivation and focus during gameplay significantly contribute to improving the technical performance of obese children, driving them to allocate extra mental resources toward coordinating their movements, thereby minimizing errors [13].

Regarding psychological responses, numerous studies underscore the importance of utilizing the Profile of Mood States (POMS) and the Physical Activity Enjoyment Scale (PACES) to evaluate affective states among OCs during physical education sessions [14,43–45]. Engaging in physical education sessions with VE has been found to positively correlate with individuals’ motivation to enhance their affective disposition through active participation in games [39]. The current study suggests that PGs with VE elicit greater enjoyment and foster a more positive mood compared to those without. These findings emphasize the influence of motivational factors on experiencing positive emotions during physical education sessions. Physical enjoyment is a positive emotional response that connects happiness to physical activity [14,43,45,46]. Moreover, this investigation demonstrates that PGs with VE elicit higher levels of physical enjoyment and promote a more positive mood state compared to those without. Our study revealed that verbal encouragement has a beneficial impact on enhancing physical enjoyment, as evidenced by a significant increase in PACES scores.

In the context of PGs, the presence of VE plays a significant role in eliciting favorable emotional reactions towards the activity, substantially contributing to engagement in physical exercise. It is hypothesized that motivational elements, coupled with VE from PET, account for the heightened sense of enjoyment experienced during PGs with VE. This finding aligns with previous research by [47], suggesting that motivation within PET enhances students’ enjoyment and promotes positive behavior. Hence, the motivation provided by teachers correlates with favorable emotional responses to physical exertion, serving as a pivotal factor in encouraging participation in the game.

Furthermore, integrating the TGFU approach into the framework of this study is particularly relevant. TGFU emphasizes understanding and applying tactical concepts in games, which aligns with our findings on VE. By fostering an environment where children are encouraged to think critically about their gameplay, PETs can enhance both technical skills and enjoyment. This approach not only supports skill development but also encourages children to engage more deeply with the game, enhancing their overall experience and motivation. Incorporating TGFU principles alongside VE can create a holistic learning environment that empowers children to improve their technical abilities while enjoying the process.

By employing strategies such as VE and the TGFU approach, PETs can establish a positive motivational atmosphere that enhances training enjoyment. All 16 participants in the current research favored PGs with VE over those without, reinforcing the idea that enjoyment derived from PET is influenced not only by the game itself but also by factors such as the type of training, teaching methods, and the motivation and satisfaction of the participants [13,14].

Mood state responses play a crucial role in determining the performance of OCs. This study noted a significant difference in POMS scores (TMD and vigor) between the PGs with VE and those without. This indicates that VE leads to a more positive mood among OCs. The findings presented in this study are consistent with prior research demonstrating the significant influence of VE on fostering a positive mood state among students during physical education classes [13,14]. These findings align with recent research [15], which highlighted that VE during small-sided soccer games produces a positive mood compared to sessions without such encouragement [42]. Previous studies have established a link between mood state, PET intervention, exercise modality, and motivation among students in physical education sessions. Our results suggest that VE can further enhance this mood state. This conclusion is supported by research indicating that VE during physical activity fosters a positive mood by decreasing TMD and increasing vigor [26]. These findings suggest that targeted ball training leads to a rise in affirmative expressions and an enhanced positive emotional state. This aligns with prior studies demonstrating the impact of VE on mood responses during physical activity [13,26].

The current study underscores the significant impact of PET behavior on OCs, highlighting the relationship between VE and performance. Research conducted by [13] revealed that the passive role of PET resulted in a decrease in psychophysiological responses among obese students and a deterioration in the motivational environment, leading to emotional decline. Conversely, the findings suggest that OCs experience greater enjoyment during workouts and increased motivation and perceive their efforts as more significant when guided by a PET who provides positive encouragement. This highlights the crucial importance of a constructive teaching style, such as providing positive feedback, in fostering optimal performance in obese students.

Several limitations should be noted when interpreting the results of this study. First, the small sample size limited the robustness of the findings due to challenges in recruiting obese children, as few were present in classes taught by the same teacher. Second, the study focused exclusively on male participants under 14 years old, which suggests that future research should include diverse groups across different sexes and ages to gain a broader understanding of physiological and psychological responses. Additionally, variations in game conditions—such as the number of ball passes, pitch size, and use of goalkeepers—were not explored, which could have provided further insights. Lastly, while the study effectively examined the impact of verbal encouragement, it did not measure key psychological factors like confidence and motivation, which could enhance understanding of their roles in engagement and performance.

## Conclusion

The findings of this study highlight the critical role that VE plays in PGs for enhancing technical skills, fostering a positive mood, increasing perceived enjoyment, and improving technical proficiency among OCs. VE is highly recommended for usage by PETs, particularly in classes where the goals are higher intensity levels, technical aspects, and positive feelings. Therefore, VE plays a pivotal role in enhancing the intensity of gameplay, refining technical skills, and fostering positive emotional states among OCs participating in physical education sessions.

### Practical applications

This study offers valuable insights for Physical Education Teachers (PETs) by demonstrating the significant impact of verbal encouragement (VE) on performance and affective responses among obese children in real-world training settings. VE fosters a supportive motivational climate, enhancing student morale while promoting technical skills such as passing accuracy and engagement during games. PETs should consciously integrate VE into their teaching strategies, especially when aiming for maximal effort and technical proficiency. Additionally, employing the Teaching Games for Understanding (TGFU) approach can further enrich the learning experience, helping children develop both tactical understanding and a positive attitude toward physical activity. Prioritizing VE and TGFU can lead to improved outcomes and greater enjoyment in physical education for obese children.

## Supporting information

S1 File Datasheet. All relevant data are within the paper and its Supporting Information Files.
(XLSX)
